# Functional Evolution and Modular Behaviour in the Carnivoran Mandible

**DOI:** 10.1093/icb/icag158

**Published:** 2026-08-26

**Authors:** G Sansalone, C Serio, G Girardi, S Castiglione

**Affiliations:** Department of Life Sciences, University of Modena and Reggio Emilia, Via Campi 213/D, 41125 Modena, Italy; Function, Evolution and Anatomy Research Lab, Zoology Division, School of Environmental and Rural Science, University of New England, Armidale, 2350, NSW, Australia; Department of Earth Sciences, Environment and Resources DiSTAR, University of Naples Federico II, Via Cintia, 26, 80126 Napoli, Italy; Department of Earth Sciences, Environment and Resources DiSTAR, University of Naples Federico II, Via Cintia, 26, 80126 Napoli, Italy; Department of Earth Sciences, Environment and Resources DiSTAR, University of Naples Federico II, Via Cintia, 26, 80126 Napoli, Italy

## Abstract

The relationship between form and function holds a central spot in evolutionary biology, as the changes in such relationship through time, are likely to deeply influence how organisms adapt to the surrounding environment. However, anatomical structures may be interpreted as a compound of so-called “modules” which may respond differently to similar biomechanical demands. To understand the extent of how the form of a modular structure relate to biomechanical performance, we combined modern 3D shape analysis with phylogenetic comparative methods, to determine whether evolutionary rates of shape are correlated with evolutionary rates of different functions. Here, we quantified the shape of 91 mandibles belonging to 67 species of eutherian carnivores and used three distinct performance metrics such as canine bite force, mechanical advantage of temporalis and masseter muscles. Results showed a large decoupling of evolutionary rates of shape and function, suggesting that many-to-one mapping of form and function plays a relevant role in obscuring a direct relationship between form and function rates of evolution. However, when we performed a robust phylogenetic regression, we found a significant relationship between evolutionary rates of mandibular shape and the mechanical advantage of the temporalis muscle. This suggests that the evolution of the mandible may show a modular behavior, hinting that different regions of the same structure may evolve at different paces and hence display different degree of correlation. In conclusion, our results highlight that even simpler anatomical structures are likely to display a mosaic evolution rather showing a direct, one-to-one, relationship between form and function.

## Introduction

Determining how organisms adapt to environmental conditions remains one of the most fascinating questions in evolutionary biology. Central to this endeavor is the relationships between form (e.g., the shape and size) and function (e.g., how it performs within the physical environment) of anatomical structures (Wainwright 2005). Establishing the adaptive significance of a phenotypic feature requires understanding the way it works (Losos 1990). Such knowledge clarifies how phenotypic evolutionary changes enhance or constrain ecological competence and how features evolve in response to selective pressures (Arnold 2003). However, most phenotypes can be composed by several traits and influenced by both genetic and environmental factors (Lande 1979; Nouailhetas Simon et al. 2025). Differences between populations may arise due to developmental plasticity such as growth rate changes (Piras et al. 2011), and/or to distinct biotic interactions (competition, resource use) linked to geographical variation at different scales (Martin and Wainwright 2013). Classical examples include Darwin finches and *Anolis* lizards from Caribbean islands where phenotypic diversification has been shaped by the pressure provided by interspecific competition and ecological opportunity (Losos 2011; Burress and Muñoz et al. 2023). Furthermore, recent approaches have emphasized the importance of evaluating performance within a morphometric context to better understand an organism’s success in complex environments (Losos 1990; Polly et al. 2016; Dickson et al. 2021). However, many morphological systems have multiple functional roles, and understanding how morphology differentially affect the performance within distinct functional scenarios can be difficult (Moen 2019). Here, anatomical structures subject to concurrent functional demands may experience trade-offs due to the inability to simultaneously optimize multiple functions. Trade-offs may affect rates and direction of morpho-functional evolution depending on which behavior mostly affect the fitness of an organism (Moen 2019; Sansalone et al. 2024). Nonetheless, multiple morphological traits may affect a single functional output (many-to-one mapping; Losos 2011; Muñoz et al. 2017; Moen 2019). The resulting evolutionary functional redundancy may, on one side, promote morphological diversification, on the other weaken the relationship between form and function (Wainwright et al. 2005; Muñoz et al. 2017).

The identification of straightforward relationship between form and function may be further obscured by the inherent modularity of almost all biological structures (Goswami et al. 2014; Felice et al. 2018). Given that anatomical structures can vary in a semi-independent fashion, minor morphological changes in a single module can disproportionately affect the biomechanical performance of the entire structure. Conversely, substantial morphological variation in one region can have a negligible effect on the overall performance (Sansalone et al. 2024; Salcido and Polly 2025).

Implicitly, this modularity suggests that evolutionary rates of form and function may be largely decoupled. Since evolutionary rates provide a reliable measure of selective magnitude (independent of lineage age and phylogenetic history), studies of natural selection frequently prioritize rate analysis over phenotypes (Uyeda et al. 2011; Baker et al. 2015). Under a modular framework, decoupling between rates of form–function arises when variation in a single module outpaces that of the entire structure, or when rapid functional shifts occur without proportional morphological change. Reasons for such pattern include the emergence of “key innovations” (novel traits capable of expanding the ecological niche of a clade; Miller et al. 2023).

Alternatively, a strong association between evolutionary rates may arise if multiple traits evolve in a correlated fashion in response to shared selective regimes (Arnold et al. 2001; Revell and Collar 2009). Identifying these association may provide a detailed indication of how complex structures evolve.

In this context, the mammalian mandible is an ideal biomechanical system to test such hypotheses as it is a relatively simple structure (composed by the single dental bone) with a relatively simple mechanical basis. Furthermore, the carnivoran mammals represent an excellent model to study the relationship between form and function, as they display a wide array of morphological and dietary diversity (Tseng 2013; Sansalone et al. 2024).

In the recent decades, multiple studies directed their efforts to provide solid frameworks to combine modern statistical shape analysis (via geometric morphometrics) and biomechanical metrics within a defined evolutionary scenario (Meloro et al. 2011; Polly et al. 2016; Raia et al. 2018; Polly 2020; Sansalone et al. 2024). Here, building upon these frameworks, we test specific hypotheses about the evolutionary dynamics of the relationship between mandible form and function in carnivoran mammals. Specifically, we address two main questions: (1) Is there a correlation between evolutionary rates of the mandible shape and its performance metrics (e.g., bite force and masticatory muscles mechanical advantage)? (2) Does the evolution of the mandible shape depend on the evolution of its performance?

## Material and methods

### Sample

The study was conducted on a sample of 91 3D mandibles representing a total of 67 species belonging to the mammalian order Carnivora, 4 extinct and 63 extant (see Table S1 for full details on the sampled specimens). The CT mandibular data was imported into the segmentation software Mimics (v.21.0, Materialise) under the DICOM format. The resulting meshes were cleaned and prepared for the subsequent morphometric analyses using the software 3Matic (Materialise).

We used only the right hemimandible, virtually reflecting the left side for the species missing the right one. Sampling effort was aimed at covering the entire morphological variation of the group by including 14 different families: Canidae (10 species), Prionodontidae (1 species), Felidae (16 species), Procyonidae (3 species), Mustelidae (23 species), Mephitidae (2 species), Ursidae (5 species), Hyaenidae (3 species), Herpestidae (2 species), Ailuridae (1 species), and Eupleridae (1 species).

We downloaded a set of 1000 time-calibrated mammalian trees from https://vertlife.org/phylosubsets/ (Upham et al. 2019) and generated a maximum clade credibility tree using the function *MaxCredTree* from the R package phangorn (Schliep 2011). We randomly selected 100 trees from the set to test for phylogenetic uncertainty. The maximum clade credibility tree and the 100 phylogenies were pruned to include only the species represented in our dataset.

## Functional data collection

**Fig. 1:**
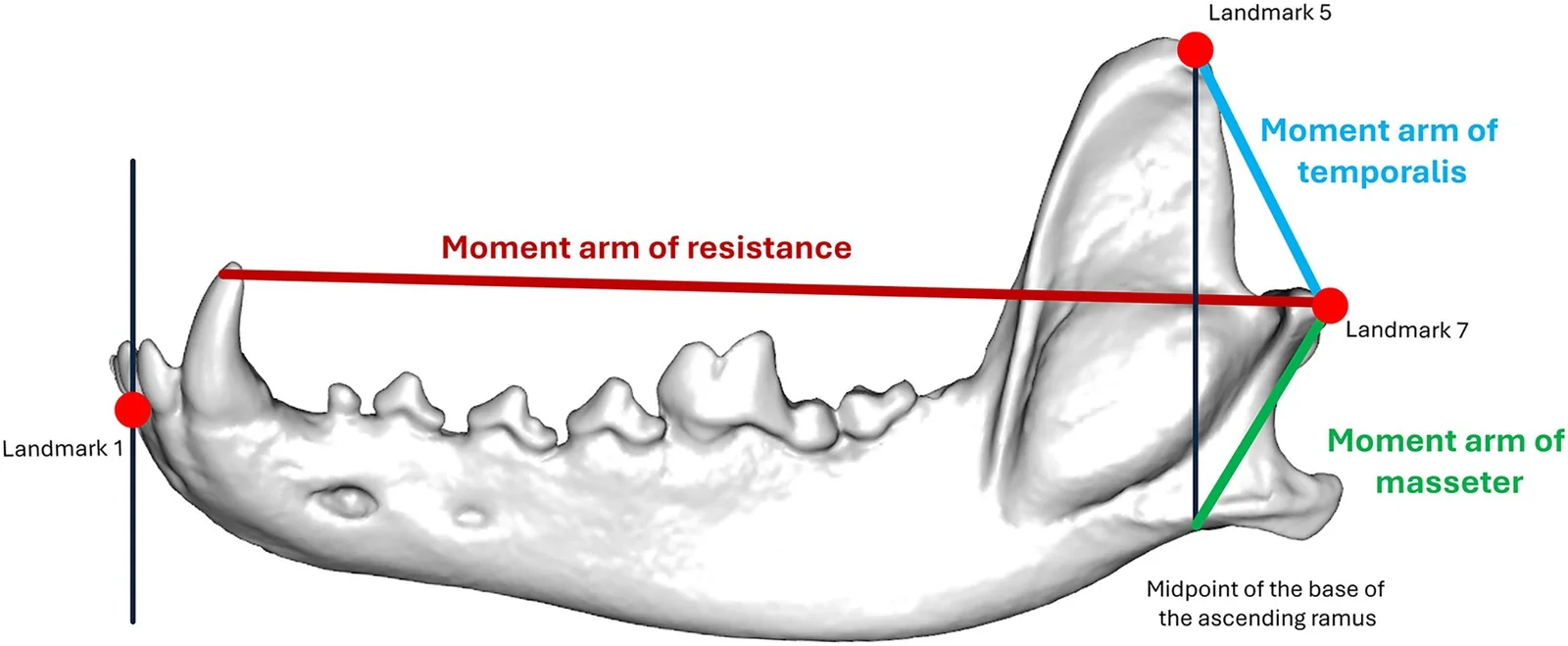
The moment arm of temporalis and masseter muscles represented on the 3D model of *Canis lupus* FW-2415.

We derived from literature three functional measurements: Anterior Bite Force (ABF), Mechanical Advantage (MA) of the temporalis, and Mechanical Advantage of the masseter (Fig. 1; see Table S2 and Supplementary Information for full details on the data and literature). ABF data were derived from literature, mainly from Sakamoto et al. (2019). Then, we integrated the missing species using data only from reliable sources that employed the dry skull method or FEA simulation in keeping with the same type of data collected form Sakamoto et al. (2019). When multiple measurements were available for the same species, we used the measurements average (Christiansen and Wroe 2007; Damasceno et al. 2013). ABF indicates bite strength at the front of the mouth, specifically quantifying the force exerted by the canines (see Sakamoto et al. 2019, for more detail on the definition). The MA of temporalis and masseter muscles (the main muscles involved in the biting process) is measured as the ratio between the muscle’s moment arm divided by the moment arm of resistance, measured at the canine (Fig. 1, to this end we ensure that we managed to include only specimens with a minimal degree of teeth wear and without broken canines). High MA values correlate with high muscular strength, whereas low MA values are indicative of rapid muscular contraction (Table S2). These metrics have been used to measure the adductor muscles performance in different taxa such as stem mammals (Morales-García et al. 2021), birds (Navalón et al. 2019), and rodents (Anderson et al. 2014). However, we recommend caution when interpreting results from analyses of MA values and shape data as a significant relationship between these variables may not be necessarily derived form a biological dependence but from statistical circularity (which can inflate such relationship) when the variables are measured in the same way (e.g., using the same landmarks).

### Shape analysis

**Fig. 2:**
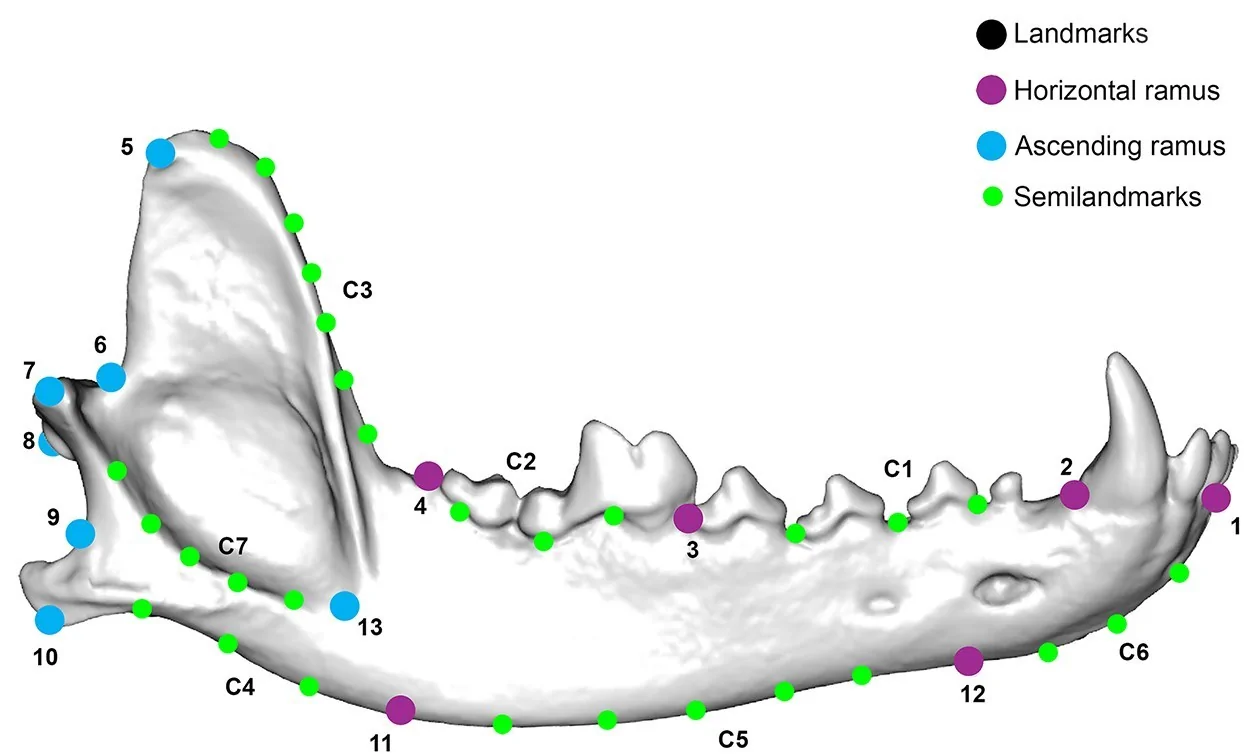
Landmarks and semilandmarks configuration applied to the mandible of *Canis lupus* FW-2415. Larger violet and blue dots represent landmarks configuration of the horizontal and ascending ramus, respectively. Smaller green dots represent semilandmarks.

We digitized 13 homologous landmarks and 29 semilandmarks (Fig. 2). Semilandmarks were used to register the curvatures of the ascending ramus, the horizontal ramus, and the masseteric fossa (Sansalone et al. 2024). Both landmarks and semilandmarks were placed on the labial side of the right hemimandible (Table S3). To account for digitization error, we assessed the magnitude of measurement error (see Supplementary Information for further details).

To remove the effect of size, rotation, and scaling by means of the Procrustes Superimposition while performing the sliding procedure we used the function *procSym* (Morpho R package; Schlager 2017). During the sliding procedure *procSym* shifts semilandmarks to make them equally spaced, reducing bias associated with manual sampling and creating correspondence among sampled species. Once removed the non-shape information by means of the Procrustes superimposition, we computed the average configuration per species. Finally, we performed the Principal Component Analysis.

### Phenotypic integration

We separated the hemimandible into two functional modules: the ascending ramus (AR herafter) holding the masticatory muscles insertion areas, and the horizontal ramus (HR hereafter) holding the teeth (Fig. 2). We measured the extent of phenotypic integration between the two mandibular modules using the function *phylo.integration* from the package geomorph. It has been recently noted that sliding semilandmarks using the minimum bending energy (BEN) approach may result in increased covariation between modules (Cardini 2019). Since we used semilandmarks in our dataset, we repeated all the following analyses using shape coordinates derived using both the minimum BEN and minimum Procrustes distances approaches to evaluate any potential discrepancy in the results. Furthermore, we repeated the following analyses performing separate Procrustes analysis on the two mandibular modules. We found no significant discrepancies when using either sliding methods, hence we present only the results obtained from the analyses performed on the shape coordinates derived after using the minimum BEN approach. On the same page, we did not find significant variations when using separate Procrustes analyses, hence we present only the results generated by performing a single Procrustes analysis on the entire shape.

### RRphylo

We estimated evolutionary rates of mandible shape evolution using the *RRphylo* method (Castiglione et al. 2018). *RRphylo* performs phylogenetic ridge regression on tree and data to estimate branch-wise evolutionary rates and ancestral characters at nodes. We then used the function *search.shift* (RRphylo R package) under the “auto.recognize” mode to automatically scan the phylogeny for evolutionary rates shifts. By default, *search.shift* selects clades ranging in size from one tenth to one half of the tree size, calculates the difference in mean absolute rates between the clade and the rest of the tree, and assesses significance via randomization. In our analyses, we set the minimum clade size to three in order to account for the small size of some families in our phylogeny (Hyaenidae *n* = 3, Procyonidae *n* = 3, Ursidae *n* = 5). Both *RRphylo* and *search.shift* were run on the PC scores of full hemimandible shape, AR and HR, as well as for functional measurements (bite force and temporalis and masseter MA). In particular, we accounted for the effect of body size on bite force, by adding the centroid size as additional predictor in *RRphylo*.

To evaluate the relationship between mandible shape (either full or modular) and functional measurements in a phylogenetically explicit context, we performed phylogenetic generalized least squares (PGLS) by means of the R function *PGLS_fossil* available in the RRphylo R package. *PGLS_fossil* accounts for rate variability across the tree by rescaling each branch according to its evolutionary rate estimated by *RRphylo*. When dealing with multivariate response variables (e.g., PC scores of mandible shape), *PGLS_fossil* estimates regression coefficients by means of the function *mvgls* in the R package mvMORPH (Clavel et al. 2015), and statistical significance is obtained by means of permutations within the function *manova.gls*.

## Robust phylogenetic regression

We tested whether phenotypic evolutionary rates at the tips for mandible shape and functional measurements were correlated by applying a robust version of PGLS regression implemented in the R package ROBRT (Adams et al. 2024. https://github.com/radamsRHA/ROBRT). We used the MM robust estimator, which is both robust and resistant to outliers (Rousseeuw and Yohai 1984).

## Testing phylogenetic uncertainty

To evaluate the robustness of our results to phylogenetic uncertainty, we performed a routine including different *overfit-* functions from the RRphylo R package. We used 100 alternative topologies sampled from the posterior distribution of 1000 trees previously downloaded.

On these phylogenies, we performed *overfitRR, overfitSS*, and *overfitPGLS*, to test the robustness of rates estimation, shifts location, and the relationship between shape and functional measurements, respectively. Then, we used them to test the uncertainty of robust phylogenetic regressions.

## Results

### Shape analysis

**Fig. 3:**
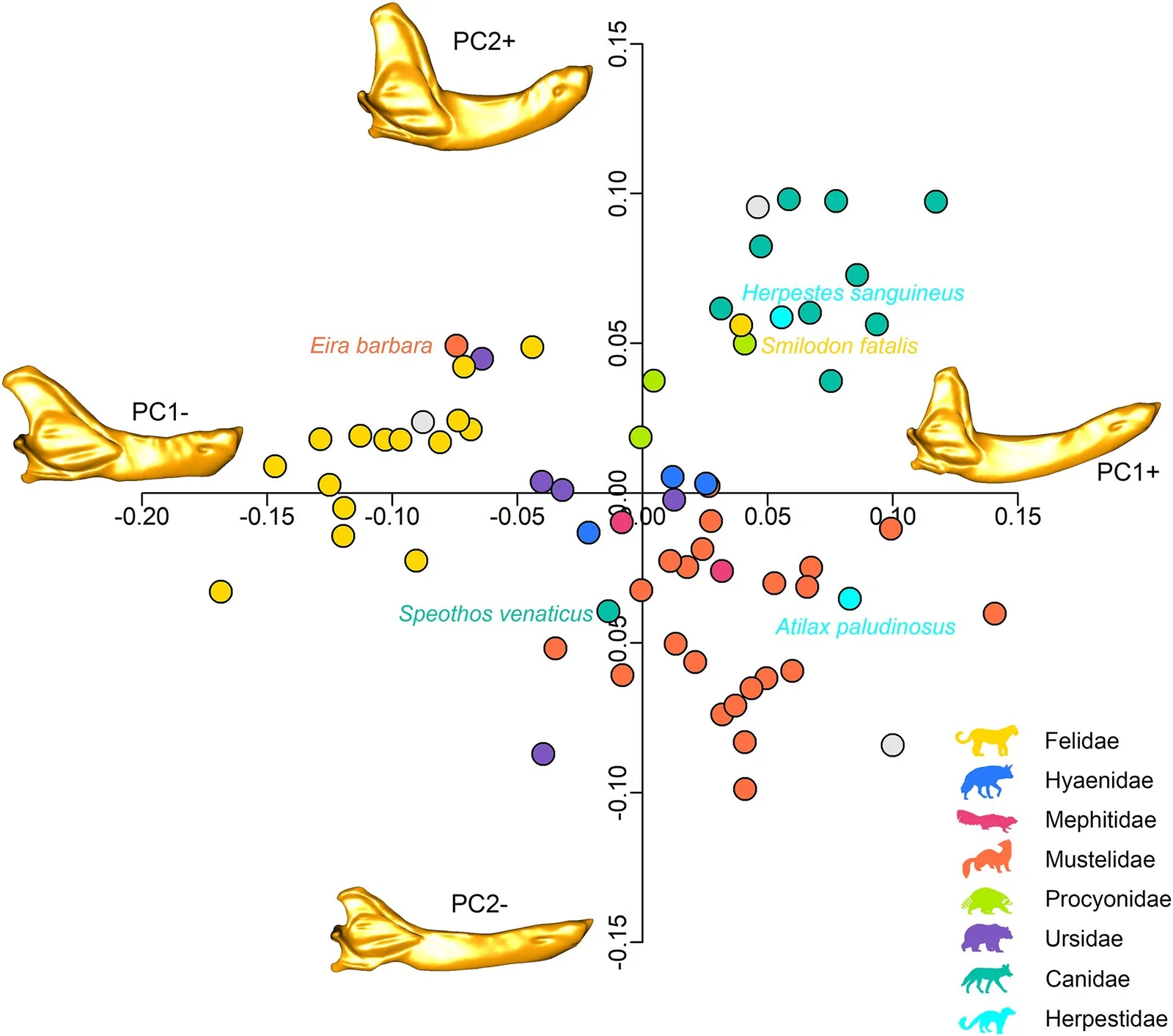
Scatterplot of the mandibular shape variation along PC1 (32.00%) and PC2 (15.07%) axes. Dots are colored according to Carnivora families; gray dots from top to bottom represent: *Prionodon linsang, Cryptoprocta ferox, Ailurus fulgens*. Deformation warping of mandibular shape along the PCs extreme were generated using *Hyeana hyaena* M USNM-1820344 as reference specimen. Silhouettes from the top: *Panthera pardus* (credits to Margot Michaud), *Hyaena hyaena* (credits to Margot Michaud), *Mephitis macroura* (credits to Mo Hassan), *Mustela putorius* (credits to Mozillian), *Procyon lotor* (credits to Gabriela Palomo-Munoz), *Ursus arctos* (credits to Margot Michaud), *Canis latrans* (credits to Patricia Holroyd), and *Herpestes sanguineus* (credits to Margot Michaud).

Mandible shape differences that have previously been inferred to relate to biomechanical function include the ventral curvature of the mandible, the orientation and thickness of the coronoid process, the thickness of the ramus, and the width of the condyle (Greaves 1985; Van Valkenburgh 1991; Van Valkenburgh et al. 2004, view Fig. 3). PCA (Principal Component Analysis) of the full shape returned 67 new axes, with PC1 accounting for 32% and PC2 for 15.07%of the total variation. The full shape morphospace is broadly structured by phylogeny, with PC1 primarily distinguishing the Feliformia (negative values) from the Caniformia (positive values; Fig. 3). In feliform morphotype, the horizontal ramus is short and thick, with an expanded angular process and a caudally oriented coronoid process (Fig. 3). In contrast, caniform morphotype displays mandible with a relatively elongated horizontal ramus with a pointy coronoid process, perpendicularly oriented with respect to the horizontal ramus (Fig. 3). At negative values of the PC2 (Fig. 3), the mandibular shape shows a long and thick horizontal ramus and a round, caudally oriented, coronoid process. Whereas at positive values of the PC2 (Fig. 3), the horizontal ramus is thick and short, with a coronoid process perpendicular to the horizontal ramus (Salcido and Polly 2025).

Despite this phylogenetic framework, several taxa deviate from their phylogenetic clusters, reflecting strong signals of functional convergence. Within the Felidae, the conicaltooth cats occupy a tight, robust cluster, whereas the sabertooth cat (here represented by *Smilodon fatalis*) shifts toward the more elongated, canid-like morphospace. This significant deviation reflects the specialized biomechanical requirements of the sabertooth bite, which requires a reduced coronoid process and an elongated mandibular geometry to facilitate a massive gape (Christiansen 2008; Chatar et al. 2024). Also, the Herpestidae deviates from the phylogenetic cluster of feliforms. *Herpestes sanguineus* overlaps with the canid morphospace region, likely due to an elongated mandible that increases jaw-closure velocity for capturing small, agile vertebrates. Whereas *Atilax paludinosus* converges with the aquatic-foraging mustelid (otters) morphotype along positive PC1 values. *Atilax* and otters share elongated mandible reflecting a transition toward semi-aquatic foraging. Hyaenidae also overlaps with both Canidae and Ursidae. This placement is possibly driven by a mandibular tooth-row that is more elongated than that of typical feliforms, combined with a degree of structural robusticity required for bone-cracking. Among Caniformia, the Mustelidae family exhibits the highest morphological disparity in the dataset, reflecting their diverse ecological niches. While most mustelids possess elongated mandibles for rapid predation, *Eira barbara* represents a functional outlier by converging with the felid morphotype. Its shorter, deeper mandible provides a higher mechanical advantage for an opportunistic diet spanning fruit to small mammals, contrasting with the slender mandible of the other mustelids (Perini and Sicuro 2025). Finally, the position of the canid *Speothos venaticus* toward negative PC1 values reflects in the short mandible corpus due to the absence of the entoconid on the inferior carnassial molar associated with its hypercarnivorous diet.

PCA computed on the two modules returned 60 and 66 new axes describing the shape variation of the horizontal and ascending ramus, respectively. PC1 and PC2 of the horizontal ramus account for 68.91% of the total shape variance, while 48.06% of the total shape variation of the ascending ramus. The horizontal and the ascending ramus morphospace also distinguish between the Feliformia and Caniformia morphotypes along the PC1 axis (Figs S1, S2). To investigate evolutionary shifts of the full shape, horizontal and ascending ramii, we used 19 PCs explaining ~96% of the full shape variance.

### RRphylo and PGLS

**Fig. 4:**
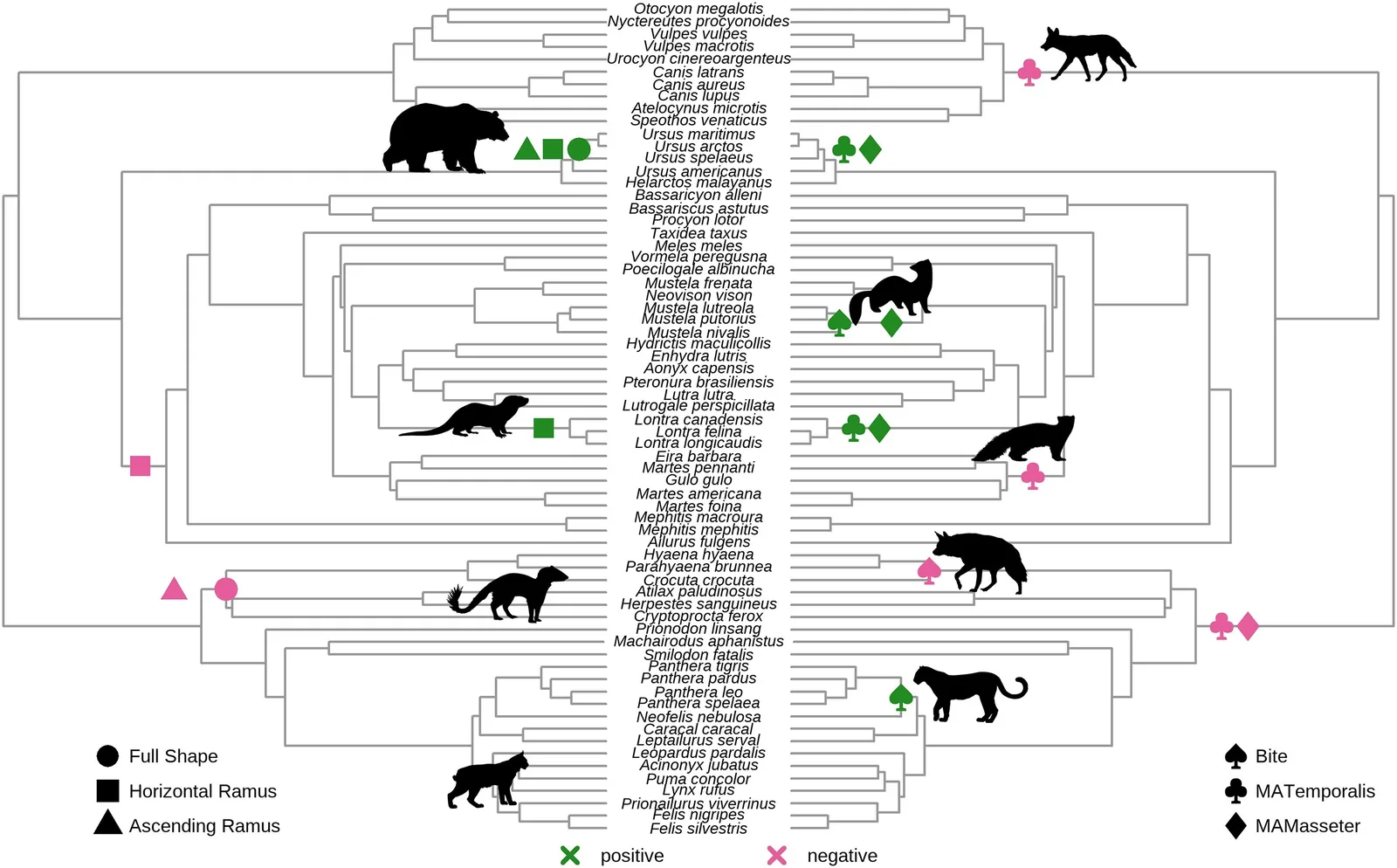
Carnivora phylogenetic tree showing shifting clades resulting from *search.shift* analyses applied on the rates of hemimandible shape (left panel) and on the rates of functional measurements (right panel). Green and pink symbols represent positive and negative shifts, respectively. MA: mechanical advantage. Silhouettes, from the top of the figure: *Canis latrans* (credits to Patricia Holroyd), *Ursus arctos* (credits to Margot Michaud), *Mustela putorius* (credits to Mozillian), *Lontra felina* (credits to Margot Michaud), *Martes foina* (credits to Ferran Sayol), *Hyaena hyaena* (credits to Margot Michaud), *Herpestes sanguineus* (credits to Margot Michaud), *Panthera pardus* (credits to Margot Michaud), *Lynx rufus* (credits to Margot Michaud).

By applying *search.shift* under “auto-recognize” mode on full and modular shape rates, we identified three focal clades (Table 1, Fig. 4). The clade including *Ursus maritimus, U. spelaeus*, and *U. arctos* (i.e., genus *Ursus* excluding *U. americanus*) shows a significant positive shift, very strongly supported (>95%) for both full and modular mandible shape data. Negative shifts in full shape and AR shape were detected for Herpestoidea (13% and 43% support, respectively). Musteloidea exhibits a significant negative shift in HR shape, strongly supported by uncertainty analyses (>75%). A positive shift regarding HR only was found in the genus *Lontra*, though it was poorly supported (25%).

**Table 1:** Results of search.shift and overfit on mandible shape evolutionary rates.

	Full shape			Horizontal ramus			Ascending ramus		
Shifting clade	Rate difference	*P*-value	Overfit	Rate difference	*P*-value	Overfit	Rate difference	*P*-value	Overfit
Musteloidea				-0.002	0.013	78%			
Herpestoidea	-0.004	0.022	13%				-0.003	0.024	43%
Genus *Lontra*				0.008	0.976	25%			
Genus *Ursus*	0.041	1.000	100%	0.023	1.000	99%	0.036	1.000	100%

Full shape: evolutionary rates of the total shape of the hemimandible; Horizontal Ramus: evolutionary rates of the shape of the horizontal ramus; Ascending Ramus: evolutionary rates of the shape of the ascending ramus. The shifting clade of the genus *Ursus* includes only *Ursus maritimus, U. arctos*, and *U. spelaeus*. Rate difference: difference of mean absolute rates for the clade and for the rest of the tree; Overfit: percentage of simulations returning significant shift.

The results of *search.shift* performed on the rates of bite force computed by accounting for the effect of centroid size show two positive for the genus *Mustela* (excluding *M. frenata*) and the *Pantherini* tribe (100% and 60% support, respectively), and a well supported negative shift for Hyaenidae (89%, Table 2, Fig. 4). The same analysis on temporalis MA identified two positive and well-supported shifts for the genus *Lontra* and the genus *Ursus* excluding *U. americanus* (78% and 98% support, respectively), and three negative, strongly supported, shifts for the subfamily Guloninae, the family Canidae and the suborder Feliformia (all > 95% support, Table 2, Fig. 4). Finally, *search.shift* applied to masseter MA produced three significant positive shifts for the genus *Mustela* (excluding *M. frenata*), the genus *Lontra*, and the genus *Ursus* excluding *U. americanus* (87%, 97%, and 60% support, respectively) and a negative shift for Feliformia (77%, Table 2, Fig. 4).

**Table 2:** Results of *search.shift* and *overfit* on evolutionary rates of bite force and mechanical advantage of muscles.

	Anterior bite force			MATemporalis			MAMasseter		
Shifting clade	Rate difference	*P*-value	Overfit	Rate difference	*P*-value	Overfit	Rate difference	*P*-value	Overfit
Genus *Mustela*	0.039	1.000	100%				0.003	0.993	87%
Genus *Lontra*				0.015	0.994	78%	0.005	1.000	97%
Guloninae				-0.002	0.002	99%			
Genus *Ursus*				0.022	1.000	98%	0.003	0.997	60%
Canidae				-0.002	0.001	99%			
Feliformia				-0.002	0.002	100%	-0.0004	0.019	77%
Pantherini	0.020	0.993	94%						
Hyenidae	-0.014	0.009	73%						

Anterior bite force: evolutionary rates of anterior bite force computed by accounting for the effect of centroid size. MATemporalis: mechanical advantage of temporalis; MAMasseter: mechanical advantage of masseter. The genus *Mustela* includes all *Mustela* species exclusive of *M. frenata*; the genus *Ursus* includes only *U. maritimus, U. arctos*, and *U. spelaeus*; Rate difference: difference between mean absolute rates for the clade and for the rest of the tree; Overfit: percentage of simulations returning significant shift.

PGLS analyses performed on full hemimandible shape and mandible modules revealed that mandibular shape does not significantly predict functional performance across the phylogeny (Table S4), suggesting a potential macroevolutionary decoupling between form and function.

Robust regression between hemimandible shape rates, either full or modular, and the mechanical advantage of temporalis muscle returned a positive and significant slope, though poorly supported (<15%, Table S5). A positive significant slope was also found for the regression between the rates of AR shape versus the rates of masseter MA, but the support was statistically negligible (4%). Other robust regression analyses yielded no significant results (Table S5).

## Discussion

Anatomical structures are generally thought to be shaped by evolution to perform specific tasks (Schwenk 2000). As noted previously, a straight, ono-to-one relationship between form and function is only ideally expected when investigating how biological systems interact with their physical environment (Wainwright et al. 2005; Losos 2011). As a matter of facts, we did not find a significant association between total or modular mandibular shape and our functional metrics (see Table S4). This suggests that the carnivoran mandible exhibits a macroevolutionary decoupling, where morphological and functional dynamics operate at different rates and magnitudes.

This decoupling may be a consequence of multiple factors, the first being the many-to-one mapping of form to function, where different morphologies realize similar functional outputs (Losos 2011; Munoz 2019; Sansalone et al. 2024). This pattern is particularly evident in our dataset, where taxa showing highly distinct morphologies and ecologies also display similar functional outputs. For example, large hypercarnivorous felids (e.g., *Panthera leo* and *P. tigris*) display bite force values comparable to the polar bear (*Ursus maritimus*), despite their different mandibular architectures and ecological specializations (see Fig. 3). However, it must be stressed out that polar bears display larger size than large hypercarnivorous felids. A similar scenario is observed in musteloid carnivores, which maintained a broadly conserved mandibular morphotype (see Fig. 3), despite displaying a high variability in terms of biomechanical performance (Prevosti et al. 2012; Law et al. 2018). Such patterns indicate a non-linear relationship between form and function changes per unit time, where minor morphological adjustments may produce large functional shifts, or rapid morphological changes may result in negligible functional modification (Tseng and Flynn 2018; Muñoz 2019; Sansalone et al. 2024). Another confounding factor can be represented by the force-velocity trade-off inherent to the mammalian mandible. Here, the mandible cannot be simultaneously optimized for both strength and velocity, and one function can only be optimized at the expense of the other (Polly et al. 2016). However, the optimization of one function may come at a higher evolutionary cost compared to the other, substantially narrowing the range of possible shapes to achieve a specific functional output, whereas multiple traits combinations are available to optimize the other (Sansalone et al. 2024). Also, factors other than purely biomechanical ones, may play a role in the evolutionary form-function decoupling we observe, as exemplified by pantherine felids, where a positive shift in bite force does not match a shift in mandible shape (see Fig. 4). Here, it is possible that the shape of the mandible in large hypercarnivorous felids might be more influenced by the need of acquiring a larger gape (to subdue larger preys), in turn obscuring a direct relationship with bite force (Slater and Van Valkenburgh 2009). Furthermore, beyond biomechanical efficiency, the mammalian mandible is a poly-functional structure influenced by sensory roles, social behavior, and mating strategies (Meloro and O’Higgins 2011). These competing selective pressures may obscure any potential coupling between form and function.

However, our analyses evidenced a notable exception to macroevolutionary decoupling in the family Ursidae. Ursids experienced morphological modifications in their cranio-mandibular system in order to modify their feeding strategies to adapt to their relative environments (e.g., durophagy, herbivory and carnivory), accompanied by rapid, coupled changes in functional outputs (see Fig. 4), as noted by previous analyses of skull and mandible shape rates of evolution (Sansalone et al. 2024; Salcido and Polly 2026).

Our detection of a significant (though poorly supported; Table S5) relationship between the evolutionary rates of shape and mechanical advantage of temporalis muscle suggests that certain anatomical regions remain tightly integrated despite the general decoupling of the mandible. This is likely a consequence of the direct relationship between the orientation, size, and origin point on the skull of the temporalis muscle and the shape of the coronoid process, where temporalis muscle inserts. For instance, most felids display a large, caudally oriented coronoid process that increase the moment arm of the temporalis muscle, thereby maximizing muscle force and relative bite force (Morales-Garcia et al. 2021). In contrast, the slender and perpendicularly oriented coronoid process in canids suggests a reduced role of the temporalis in the feeding process. This has been demonstrated in other empirical studies where the temporalis muscle has been shown to have lower performance in canids (Penrose et al. 2020; Hartstone-Rose et al. 2022; Salcido and Polly 2026). Collectively, these results suggest that functional changes in the temporalis muscle are intrinsically linked to morphological modifications in the shape of coronoid process. This indicates that while the mandible, as a whole, exhibits macroevolutionary decoupling, specific biomechanical interfaces (such as the muscle insertion points on the ascending ramus) remain tightly integrated.

These findings should encourage future investigations to account for the modular behavior of the carnivoran mandible (see also Salcido and Polly 2026 for further discussion on this topic). This structure is organized into two distinct functional modules (the horizontal ramus holding the teeth row and the ascending ramus allowing the insertion of the masticatory muscles) which are governed by independent genetic and developmental controls (Atchley and Hall 1991). This modular autonomy likely allows each region to respond to different selective pressures, facilitating the macroevolutionary decoupling observed in our results. In this sense, evolutionary mosaicism may be invoked as an important driver behind the functional diversity of carnivores (Law et al. 2024; Salcido and Polly 2026). Future investigations should be aimed at including also non-carnvivoran taxa to understand whether a similar pattern can be identified also in other mammalian clades.

In conclusion, the evidence for a coupling between evolutionary rates of form and function remains elusive. Our results align with a growing body of literature suggesting that a weak association between morphological and functional rates may be a general feature in the evolution of different anatomical structures (Collar and Wainwright 2006). Even a structurally simple bone such as the mammalian jaw (composed by a single dental bone) can show complex modular dynamics. Future investigations should focus on these modular architectures, testing for the functional effects of modularity and integration to uncover the ways in which anatomical structures facilitate organism’s evolution.

## Supplementary Material

icag158_Supplemental_File

## Notes

From the symposium “Integrating integration: How cellular mechanisms of trait covariation shape evolutionary patterns of phenotypic integration” presented at the annual meeting of the Society for Integrative and Comparative Biology, January 3-7th , 2026.
