# Supplementary material for: Functional Evolution and Modular Behaviour in the Carnivoran Mandible
Source: Integr Comp Biol. 2026 Aug 26;66:icag158. doi: 10.1093/icb/icag158 (PMC13613542; doi:10.1093/icb/icag158)
Supplement: Supplementary file 1 [file icag158_supplemental_file.docx]

**Supplementary Information**

**Supplementary Tables and Figures**

| **Species** | **Family** | **ID Code** |
| --- | --- | --- |
| *Ailurus fulgens* | Ailuridae | 1911.10.18.1 |
| *Atelocynus microtis* | Canidae | FMNH-60674 |
| *Canis aureus* | Canidae | MRIPAS-170857 |
| *Canis latrans* | Canidae | UV-2327 |
| *Canis latrans* | Canidae | UV-959 |
| *Canis latrans* | Canidae | UWYMV-2860 |
| *Canis lupus* | Canidae | TMN-M-1709 |
| *Canis lupus* | Canidae | FW-2415 |
| *Nyctereutes procyonoides* | Canidae | USNM-255530 |
| *Nyctereutes procyonoides* | Canidae | l-ahr |
| *Otocyon megalotis* | Canidae | USNM-429132 |
| *Otocyon megalotis* | Canidae | USNM-429129 |
| *Speothos venaticus* | Canidae | UF-19126-5M |
| *Urocyon cinereoargenteus* | Canidae | NHMUK-1850 |
| *Vulpes macrotis* | Canidae | L-AHR-0003711783_2 |
| *Vulpes vulpes* | Canidae | FW-1177 |
| *Cryptoprocta ferox* | Eupleridae | NHMUK-1968 |
| *Acinonyx jubatus* | Felidae | AMNH-119657 |
| *Acinonyx jubatus* | Felidae | FMNH-29635 |
| *Caracal caracal* | Felidae | l-ahr |
| *Felis nigripes* | Felidae | l-ahr |
| *Felis silvestris* | Felidae | 010608-SW |
| *Felis silvestris* | Felidae | 080521-1t01 |
| *Leopardus pardalis* | Felidae | I-AHR_2 |
| *Leptailurus serval* | Felidae | l-ahr |
| *Lynx rufus* | Felidae | UCLA-10118 |
| *Lynx rufus* | Felidae | UV-155 |
| *Lynx rufus* | Felidae | OUVC-9576 |
| *Machairodus aphanistus* | Felidae | NHMUK-PV-OR-37356 |
| *Neofelis nebulosa* | Felidae | UF-26153 |
| *Panthera leo* | Felidae | 190706-CM01 |
| *Panthera leo* | Felidae | MVZ-117849 |
| *Panthera pardus* | Felidae | LACM-11704 |
| *Panthera pardus* | Felidae | IMNH-2372 |
| *Panthera spelaea* | Felidae | IMNH |
| *Panthera tigris* | Felidae | MVZ-189634 |
| *Prionailurus viverrinus* | Felidae | NHMUK-1860 |
| *Puma concolor* | Felidae | UV-4117 |
| *Puma concolor* | Felidae | LACM-87430 |
| *Smilodon fatalis* | Felidae | AMNH-14349 |
| *Smilodon fatalis* | Felidae | Lige |
| *Smilodon fatalis* | Felidae | UW46981 |
| *Atilax paludinosus* | Herpestidae | NHMUK_1867 |
| *Herpestes sanguineus* | Herpestidae | USNM-539732 |
| *Crocuta crocuta* | Hyaenidae | IMNH-MVZ-165169 |
| *Crocuta crocuta* | Hyaenidae | IMNH-MVZ-165160 |
| *Hyaena hyaena* | Hyaenidae | USNM-182034 |
| *Parahyaena brunnea* | Hyaenidae | MM91_2 |
| *Mephitis macroura* | Mephitidae | J4240_53um |
| *Mephitis mephitis* | Mephitidae | l-ahr |
| *Aonyx capensis* | Mustelidae | CMNH-VZ-17620 |
| *Eira barbara* | Mustelidae | l-ahr_CTR_2 |
| *Enhydra lutris* | Mustelidae | AMNH-M-24186 |
| *Enhydra lutris* | Mustelidae | FW-1506 |
| *Gulo gulo* | Mustelidae | FW-3174 |
| *Gulo gulo* | Mustelidae | no-id_2 |
| *Hydrictis maculicollis* | Mustelidae | AMNH-M-89807 |
| *Lontra canadensis* | Mustelidae | AMNH-M-254476 |
| *Lontra canadensis* | Mustelidae | FW-6091 |
| *Lontra felina* | Mustelidae | AMNH-M-48193 |
| *Lontra longicaudis* | Mustelidae | AMNH-M-98589 |
| *Lutra lutra* | Mustelidae | AMNH-M-206592 |
| *Lutrogale perspicillata* | Mustelidae | AMNH-M-204747 |
| *Martes americana* | Mustelidae | FW-3253 |
| *Martes americana* | Mustelidae | IMNH-2311 |
| *Martes foina* | Mustelidae | no-id |
| *Martes pennanti* | Mustelidae | IMNH-760 |
| *Meles meles* | Mustelidae | NHMUK-2002 |
| *Mustela frenata* | Mustelidae | JM8543_53um |
| *Mustela lutreola* | Mustelidae | L-SP-58 |
| *Mustela nivalis* | Mustelidae | UWYMV-6544_2 |
| *Mustela nivalis* | Mustelidae | FMNH-207033 |
| *Mustela putorius* | Mustelidae | J13253_53um |
| *Neovison vison* | Mustelidae | L-SP-L1 |
| *Poecilogale albinucha* | Mustelidae | CG-1934-107 |
| *Pteronura brasiliensis* | Mustelidae | AMNH-M-98594 |
| *Taxidea taxus* | Mustelidae | LACM-45012 |
| *Vormela peregusna* | Mustelidae | JM1044_53um |
| *Prionodon linsang* | Prionodontidae | USNM303036 |
| *Bassaricyon alleni* | Procyonidae | FMNH-104935 |
| *Bassariscus astutus* | Procyonidae | MSB-Mamm-46326 |
| *Bassariscus astutus* | Procyonidae | IMNHR-2580 |
| *Procyon lotor* | Procyonidae | INHS-7241 |
| *Procyon lotor* | Procyonidae | LACM-052261 |
| *Helarctos malayanus* | Ursidae | AMNH-16580 |
| *Ursus americanus* | Ursidae | UV-4884 |
| *Ursus americanus* | Ursidae | USNM-227070 |
| *Ursus arctos* | Ursidae | USNM-98062 |
| *Ursus arctos* | Ursidae | FC30_100727 |
| *Ursus maritimus* | Ursidae | USNM-275072 |
| *Ursus spelaeus* | Ursidae | NHMW-Geo-2022-0019-0001 |

**Table S1.** The list of species used in this study and their institutional code. AMNH = American Museum of Natural History; FMNH = FIeld Museum of Natural History; IMNH = Idaho Museum of Natural History; NHMUK = Natural History Museum, United Kingdom; BSM = Museum of Southwestern Biology; UWYMV = University of Wyoming Museum of Vertebrates; TMN = Texas Memorial Museum; USNM = United State National Museum; UCLA = University of California, Los Angeles; OUVC = Ohio University Vertebrate Collection; UCMP = University of California Museum of Paleontology; NHMW = Naturhistorisches Museum Wien; LACM = Los Angeles County Museum of Natural History.

| **Species** | **Anterior Bite Force (N)** | **MATemporalis** | **MAMasseter** |
| --- | --- | --- | --- |
| *Ailurus fulgens* | 413.1 | 0.342 | 0.215 |
| *Atelocynus microtis* | 120.3 | 0.198 | 0.118 |
| *Canis aureus* | 220.7 | 0.198 | 0.118 |
| *Canis latrans* | 322.9 | 0.195 | 0.118 |
| *Canis lupus* | 563.8 | 0.203 | 0.130 |
| *Nyctereutes procyonoides* | 135.4 | 0.208 | 0.132 |
| *Otocyon megalotis* | 105.9 | 0.186 | 0.098 |
| *Speothos venaticus* | 340.8 | 0.253 | 0.133 |
| *Urocyon cinereoargenteus* | 114.1 | 0.211 | 0.105 |
| *Vulpes macrotis* | 109.5 | 0.190 | 0.093 |
| *Vulpes vulpes* | 207.4 | 0.197 | 0.100 |
| *Cryptoprocta ferox* | 290.1 | 0.298 | 0.172 |
| *Acinonyx jubatus* | 449.4 | 0.265 | 0.137 |
| *Caracal caracal* | 363.5 | 0.266 | 0.148 |
| *Felis nigripes* | 101.1 | 0.277 | 0.162 |
| *Felis silvestris* | 117.7 | 0.237 | 0.135 |
| *Leopardus pardalis* | 383.6 | 0.262 | 0.140 |
| *Leptailurus serval* | 267.2 | 0.253 | 0.131 |
| *Lynx rufus* | 232.3 | 0.247 | 0.109 |
| *Machairodus aphanistus* | 1077.4 | 0.205 | 0.115 |
| *Neofelis nebulosa* | 619.1 | 0.258 | 0.134 |
| *Panthera leo* | 1882.7 | 0.244 | 0.130 |
| *Panthera pardus* | 790.5 | 0.242 | 0.136 |
| *Panthera spelaea* | 1811.1 | 0.241 | 0.130 |
| *Panthera tigris* | 1859.4 | 0.254 | 0.128 |
| *Prionailurus viverrinus* | 311.4 | 0.223 | 0.139 |
| *Puma concolor* | 708.5 | 0.255 | 0.142 |
| *Smilodon fatalis* | 1079.1 | 0.190 | 0.106 |
| *Atilax paludinosus* | 190.4 | 0.321 | 0.175 |
| *Herpestes sanguineus* | 90.3 | 0.289 | 0.172 |
| *Crocuta crocuta* | 732.5 | 0.287 | 0.119 |
| *Hyaena hyaena* | 712.4 | 0.275 | 0.120 |
| *Parahyaena brunnea* | 888.2 | 0.308 | 0.132 |
| *Mephitis macroura* | 88.6 | 0.337 | 0.181 |
| *Mephitis mephitis* | 146.6 | 0.350 | 0.173 |
| *Aonyx capensis* | 142.4 | 0.378 | 0.117 |
| *Eira barbara* | 338.6 | 0.356 | 0.145 |
| *Enhydra lutris* | 562.1 | 0.362 | 0.151 |
| *Gulo gulo* | 350.8 | 0.374 | 0.148 |
| *Hydrictis maculicollis* | 186.4 | 0.392 | 0.167 |
| *Lontra canadensis* | 288.6 | 0.418 | 0.197 |
| *Lontra felina* | 201.8 | 0.316 | 0.155 |
| *Lontra longicaudis* | 258.2 | 0.394 | 0.176 |
| *Lutra lutra* | 295.6 | 0.395 | 0.187 |
| *Lutrogale perspicillata* | 405.2 | 0.384 | 0.167 |
| *Martes americana* | 84.8 | 0.359 | 0.128 |
| *Martes foina* | 124.6 | 0.358 | 0.129 |
| *Martes pennanti* | 226.2 | 0.363 | 0.116 |
| *Meles meles* | 210.6 | 0.293 | 0.118 |
| *Mustela frenata* | 29.1 | 0.361 | 0.146 |
| *Mustela lutreola* | 54.1 | 0.379 | 0.143 |
| *Mustela nivalis* | 24.1 | 0.400 | 0.185 |
| *Mustela putorius* | 120.8 | 0.382 | 0.136 |
| *Neovison vison* | 74.8 | 0.295 | 0.141 |
| *Poecilogale albinucha* | 37.1 | 0.315 | 0.127 |
| *Pteronura brasiliensis* | 532.12 | 0.315 | 0.154 |
| *Taxidea taxus* | 330.6 | 0.350 | 0.199 |
| *Vormela peregusna* | 28.7 | 0.299 | 0.161 |
| *Prionodon linsang* | 63.4 | 0.248 | 0.152 |
| *Bassaricyon alleni* | 117.4 | 0.284 | 0.123 |
| *Bassariscus astutus* | 109.4 | 0.274 | 0.122 |
| *Procyon lotor* | 181.3 | 0.261 | 0.132 |
| *Helarctos malayanus* | 1449.1 | 0.240 | 0.114 |
| *Ursus americanus* | 860.1 | 0.256 | 0.122 |
| *Ursus arctos* | 1347.5 | 0.257 | 0.111 |
| *Ursus maritimus* | 1561.3 | 0.201 | 0.121 |
| *Ursus spelaeus* | 1367.2 | 0.248 | 0.115 |

**Table S2.** Functional measurement. Anterior bite force is measured in Newton (N), MATemporalis: mechanical advantage of the temporalis muscle; MAMasseter: mechanical advantage of the masseter muscle.

| **A** |  |  |  |
| --- | --- | --- | --- |
| **Landmark** | **Description** |  |  |
| 1 | Most antero-dorsal point of the mandibular symphysis, middle |  |  |
| 2 | point immediately following the canine, middle |  |  |
| 3 | Most antero-dorsal point than carnaxial |  |  |
| 4 | Most posterior point on the molar row, when no teeth, most postero-dorsal point on the body of the mandible |  |  |
| 5 | Point at the tip of the coronoid process |  |  |
| 6 | Most concave point of the mandibular symphysis |  |  |
| 7 | Left end point on the mandibular condyle |  |  |
| 8 | Right end point on the mandibular condyle |  |  |
| 9 | Most ventral point of the mandibular condyle |  |  |
| 10 | Point at the tip of the angular process |  |  |
| 11 | Point perpendicular to Landmark on the inferior margin of the ramus. Middle |  |  |
| 12 | Most antero-ventral point of the mandibular symphysis, middle |  |  |
| 13 | Anterior edge of the masseteric ridge |  |  |
|  |  |  |  |
| **B** |  |  |  |
| **Curves** | **Description** | **Position** | **Number of points** |
| 1 | Medial line along the teeth row | 2 → 3 | 3 |
| 2 | Medial line along the teeth row | 3 → 4 | 3 |
| 3 | Curve starting from the base of the coronoid process to its tip | 4 → 5 | 7 |
| 4 | Ventral border of the angular process | 10 → 11 | 3 |
| 5 | Ventral border of the body of the mandible | 11 → 12 | 5 |
| 6 | Anterior curve on the symphysis, middle | 12 → 1 | 3 |
| 7 | Ventral line of the masseteric ridge | 13 → 6 | 5 |

**Table S3.** Landmark and semilandmark position and description.

|  | **FULL** | | | **HRAMUS** | | | **ARAMUS** | | |
| --- | --- | --- | --- | --- | --- | --- | --- | --- | --- |
|  | **Pillai stat** | **p-value** | **overfit** | **Pillai stat** | **p-value** | **overfit** | **Pillai stat** | **p-value** | **overfit** |
| **ABF** | 0.383 | 0.106 | 0% | 0.389 | 0.105 | 0% | 0.357 | 0.187 | 0% |
| **ABFvsCS residuals** | 0.213 | 0.855 | 0% | 0.249 | 0.693 | 0% | 0.175 | 0.949 | 0% |
| **MAT** | 0.350 | 0.221 | 0% | 0.285 | 0.529 | 0% | 0.374 | 0.124 | 0% |
| **MAM** | 0.249 | 0.707 | 0% | 0.212 | 0.838 | 0% | 0.269 | 0.579 | 0% |

**Table S4.** Results of *PGLS_fossil* and *overfit*- on mandibular shape (either full or modular) versus bite force/mechanical advantage (for either temporal or masseter). ABF: anterior bite force; ABFvsCS residuals: residuals of ABF versus centroid size regression; MAT: mechanical advantage of temporal muscle; MAM: mechanical advantage of masseter muscle. Pillai statistic: refers to the Pillai-Bartlett trace statistic; overfit: percentage of simulations returning significant p-value.

| **A** |  |  |  |  |  |  |  |
| --- | --- | --- | --- | --- | --- | --- | --- |
|  |  | **Full shape** | | | | | |
|  |  | **estimate** | **CI 2.5** | **CI 97.5** | **p-value** | **overfit** | |
|  |  |  |  |  |  | **positive** | **negative** |
| **intercept** | **ABF** | -0.0002 | -0.005 | 0.004 | 0.927 | 0% | 0% |
|  | **MAT** | -0.00003 | -0.005 | 0.005 | 0.990 | 0% | 0% |
|  | **MAM** | -0.0002 | -0.004 | 0.004 | 0.927 | 0% | 0% |
| **slope** | **ABF** | 0.004 | -0.016 | 0.024 | 0.728 | 4% | 3% |
|  | **MAT** | 0.308 | 0.279 | 0.337 | <0.001 | 3% | 4% |
|  | **MAM** | 0.157 | -0.117 | 0.432 | 0.266 | 3% | 3% |
|  |  |  |  |  |  |  |  |
| **B** |  |  |  |  |  |  |  |
|  |  | **Horizontal ramus** | | | | | |
|  |  | **estimate** | **CI 2.5** | **CI 97.5** | **p-value** | **overfit** | |
|  |  |  |  |  |  | **positive** | **negative** |
| **intercept** | **ABF** | -0.0001 | -0.003 | 0.003 | 0.953 | 0% | 0% |
|  | **MAT** | -0.0005 | -0.004 | 0.003 | 0.753 | 0% | 0% |
|  | **MAM** | -0.00043 | -0.004 | 0.003 | 0.786 | 0% | 0% |
| **slope** | **ABF** | 0.016 | 0.002 | 0.030 | 0.031 | 6% | 8% |
|  | **MAT** | 0.147 | 0.128 | 0.166 | <0.001 | 12% | 6% |
|  | **MAM** | 0.054 | -0.144 | 0.252 | 0.593 | 6% | 6% |
|  |  |  |  |  |  |  |  |
| **C** |  |  |  |  |  |  |  |
|  |  | **Ascending ramus** | | | | | |
|  |  | **estimate** | **CI 2.5** | **CI 97.5** | **p-value** | **overfit** | |
|  |  |  |  |  |  | **positive** | **negative** |
| **intercept** | **ABF** | 0.0004 | -0.003 | 0.004 | 0.792 | 0% | 0% |
|  | **MAT** | 0.0007 | -0.002 | 0.004 | 0.680 | 0% | 0% |
|  | **MAM** | -0.00016 | -0.005 | 0.005 | 0.949 | 0% | 0% |
| **slope** | **ABF** | 0.001 | -0.014 | 0.016 | 0.917 | 5% | 6% |
|  | **MAT** | 0.280 | 0.261 | 0.299 | <0.001 | 6% | 6% |
|  | **MAM** | -0.557 | -0.756 | -0.358 | <0.001 | 4% | 5% |

**Table S5.** Results of robust regression analysis on mandibular shape (either full or modular) versus bite force/mechanical advantage (for either temporal or masseter) evolutionary rates. ABF: anterior bite force accounting for the effect of centroid size on evolutionary rates; MAT: mechanical advantage of temporal muscle; MAM: mechanical advantage of masseter muscle. CI-: confidence interval; overfit: percentage of simulations returning positive/negative significant estimates. A. Results of the full shape evolutionary rates versus bite force/mechanical advantage (for either temporal or masseter) evolutionary rates; B. Results of horizontal ramus shape evolutionary rates versus bite force/mechanical advantage (for either temporal or masseter) evolutionary rates; C. Results of ascending ramus shape evolutionary rates versus bite force/mechanical advantage (for either temporal or masseter) evolutionary rates.

**Figure S1. Scatterplot of the horizontal ramus shape variation along PC1 (45.12%) and PC2 (23.79%) axes**. **Dots are coloured according to Carnivora families; grey dots from top to bottom represent: *Prionodon linsang*, *Cryptoprocta ferox, Ailurus fulgens*. Deformation warping of mandibular shape along the PCs extreme were generated using *Hyeana hyaena* M USNM-1820344 as reference specimen. Silhouettes from the top: *Panthera pardus* (credits to Margot Michaud)*, Hyaena hyaena* (credits to Margot Michaud)*, Mephitis macroura* (credits to Mo Hassan)*, Mustela putorius* (credits to** [**Mozillian**](https://www.phylopic.org/contributors/4933820d-1790-4f95-943a-dfc56b66580a/mozillian-silhouettes)**)*, Procyon lotor* (credits to Gabriela Palomo-Munoz)*, Ursus arctos* (credits to Margot Michaud)*, Canis latrans* (credits to** [**Patricia Holroyd**](https://www.phylopic.org/contributors/8bed0daa-b901-460e-9e5c-06119bddf1ce/patricia-holroyd-silhouettes)**)*,* and *Herpestes sanguineus* (credits to Margot Michaud)*.***

**Figure S2. Scatterplot of the ascending ramus shape variation along PC1 (29.32%) and PC2 axes (18.74%)**. **Dots are coloured according to Carnivora families; grey dots from top to bottom represent: *Prionodon linsang*, *Cryptoprocta ferox, Ailurus fulgens*. Deformation warping of mandibular shape along the PCs extreme were generated using *Hyeana hyaena* M USNM-1820344 as reference specimen. Silhouettes from the top: *Panthera pardus* (credits to Margot Michaud)*, Hyaena hyaena* (credits to Margot Michaud)*, Mephitis macroura* (credits to Mo Hassan)*, Mustela putorius* (credits to** [**Mozillian**](https://www.phylopic.org/contributors/4933820d-1790-4f95-943a-dfc56b66580a/mozillian-silhouettes)**)*, Procyon lotor* (credits to Gabriela Palomo-Munoz)*, Ursus arctos* (credits to Margot Michaud)*, Canis latrans* (credits to** [**Patricia Holroyd**](https://www.phylopic.org/contributors/8bed0daa-b901-460e-9e5c-06119bddf1ce/patricia-holroyd-silhouettes)**)*,* and *Herpestes sanguineus* (credits to Margot Michaud)*.***

**Supplementary Methods**

**Measurement Error**

To quantify the digitisation error and ensure the replicability of the landmark data, a subset of 13 mandibles (Table S6) was randomly selected for re-digitisation. A single operator performed three independent digitisation trials on these specimens over three consecutive days. The magnitude of measurement error relative to biological variation was assessed via a Procrustes ANOVA to calculate the repeatability index, following the procedure described by Fruciano et al. (2016). This index allows for the partitioning of variance to confirm that the technical error is negligible compared to the interspecific morphological variation.

| **Species** | **Family** | **id code** |
| --- | --- | --- |
| *Aonyx capensis* | Mustelidae | CMNH-VZ-17620 |
| *Bassaricyon alleni* | Procyonidae | FMNH-104935 |
| *Caracal caracal* | Felidae | l-ahr |
| *Felis silvestris* | Felidae | 080521-1t01 |
| *Hyaena hyaena* | Hyaenidae | USNM-182034 |
| *Lynx rufus* | Felidae | UCLA-10118 |
| *Nyctereutes procyonoides* | Canidae | USNM-255530 |
| *Panthera leo* | Felidae | MVZ-117849 |
| *Panthera pardus* | Felidae | IMNH-2372 |
| *Poecilogale albinucha* | Mustelidae | CG-1934-107 |
| *Smilodon fatalis* | Felidae | UW46981 |
| *Ursus americanus* | Ursidae | USNM-227070 |
| *Vulpes vulpes* | Canidae | FW-1177 |

**Table S6**. The 13 species selected to test for measurement error.

**Centroid size**

We computed the centroid size for the full shape to investigate the effect of mandible size. The centroid size is the square root of the square distance between each landmark and the center of the mandible. We used the *PGLS_fossil* function (RRphylo R package) to investigate the relationships between mandible size and bite force/mechanical advantage (temporal and masseter). We run *overfitPGLS* (RRphylo R package) on 100 alternative phylogenetic tree topologies to assess the robustness of the results. Before analyses, centroid size was log_10_ transformed.

**Results**

**Measurement error**

Procrustes ANOVA revealed no significant difference between replicate digitisation trials (p = 1.0, R^2^ < 0.001). The repeatability index was found to be exceptionally high (R = 0.994), confirming that measurement error accounted for a negligible portion of the total shape variance.

**Centroid size**

*PGLS_fossil* showed centroid size is significantly related with bite force and the result is 100% supported by *overfitPGLS* (Table S7, Figure S3). By contrast, no significant results were found for mechanical advantage of temporal and masseter (Table S7).

**Figure S3.** Scatterplots of (log10) centroid size versus bite force and mechanical advantage of temporal and masseter muscles. Dots are coloured according to the Carnivora families. Silhouettes from the top: ***Panthera pardus* (credits to Margot Michaud)*, Hyaena hyaena* (credits to Margot Michaud)*, Mephitis macroura* (credits to Mo Hassan)*, Mustela putorius* (credits to** [**Mozillian**](https://www.phylopic.org/contributors/4933820d-1790-4f95-943a-dfc56b66580a/mozillian-silhouettes)**)*, Procyon lotor* (credits to Gabriela Palomo-Munoz)*, Ursus arctos* (credits to Margot Michaud)*, Canis latrans* (credits to** [**Patricia Holroyd**](https://www.phylopic.org/contributors/8bed0daa-b901-460e-9e5c-06119bddf1ce/patricia-holroyd-silhouettes)**)*,* and *Herpestes sanguineus* (credits to Margot Michaud)*.***

|  |  | **estimate** | **Std. Err.** | **t.value** | **p.value** | **overfit** |
| --- | --- | --- | --- | --- | --- | --- |
| **ABF** | **Intercept** | 0.386 | 0.359 | 1.075 | 0.286 | 2% |
|  | **Slope** | 0.918 | 0.141 | 6.512 | <0.001 | 100% |
| **MAT** | **Intercept** | 0.274 | 0.033 | 8.292 | <0.001 | 100% |
|  | **Slope** | -0.005 | 0.013 | -0.405 | 0.687 | 0% |
| **MAM** | **Intercept** | 0.166 | 0.021 | 7.857 | <0.001 | 100% |
|  | **Slope** | -0.013 | 0.008 | -1.580 | 0.119 | 0% |

**Table S7.** Results of *PGLS_fossil* and *overfit*- on centroid size versus bite force/mechanical advantage (for either temporal or masseter). Std. Err.: standard error; overfit: percentage of simulations returning significant estimates. MAT: mechanical advantage of temporal; MAM: mechanical advantage of masseter.

**References**

Christiansen, P., & Adolfssen, J. S. (2005). Bite forces, canine strength and skull allometry in carnivores (Mammalia, Carnivora). *Journal of Zoology*, *266*(2), 133–151. <https://doi.org/10.1017/s0952836905006643>

Christiansen, P., & Wroe, S. (2007). BITE FORCES AND EVOLUTIONARY ADAPTATIONS TO FEEDING ECOLOGY IN CARNIVORES. *Ecology*, *88*(2), 347–358. <https://doi.org/10.1890/0012-9658(2007)88>

Binder, W. J., & Van Valkenburgh, B. (2000). Development of bite strength and feeding behaviour in juvenile spotted hyenas (Crocuta crocuta). *Journal of Zoology*, *252*(3), 273–283. <https://doi.org/10.1111/j.1469-7998.2000.tb00622.x>

Damasceno, E. M., Hingst‐Zaher, E., & Astúa, D. (2013). Bite force and encephalization in the C anidae (M ammalia: C arnivora). *Journal of Zoology*, *290*(4), 246-254.

Fruciano, C. (2016). Measurement error in geometric morphometrics. *Development genes and evolution*, *226*(3), 139-158.

Hartstone‐Rose, A., Dickinson, E., Deutsch, A. R., Worden, N., & Hirschkorn, G. A. (2021). Masticatory muscle architectural correlates of dietary diversity in Canidae, Ursidae, and across the order Carnivora. *The Anatomical Record*, *305*(2), 477–497. <https://doi.org/10.1002/ar.24748>

Hartstone‐Rose, A., Perry, J. M. G., & Morrow, C. J. (2012). Bite force estimation and the fiber architecture of felid masticatory muscles. *The Anatomical Record*, *295*(8), 1336–1351. <https://doi.org/10.1002/ar.22518>

McHenry, C. R., Wroe, S., Clausen, P. D., Moreno, K., & Cunningham, E. (2007). Supermodeled sabercat, predatory behavior in Smilodon fatalis revealed by high-resolution 3D computer simulation. *Proceedings of the National Academy of Sciences*, *104*(41), 16010–16015. <https://doi.org/10.1073/pnas.0706086104>

Sakamoto, M., Ruta, M., & Venditti, C. (2019). Extreme and rapid bursts of functional adaptations shape bite force in amniotes. *Proceedings of the Royal Society B Biological Sciences*, *286*(1894), 20181932. <https://doi.org/10.1098/rspb.2018.1932>

Slater, G. J., & Friscia, A. R. (2019). Hierarchy in adaptive radiation: a case study using the Carnivora (Mammalia). *Evolution*, *73*(3), 524-539.

Wroe, S., McHenry, C., & Thomason, J. (2005). Bite club: comparative bite force in big biting mammals and the prediction of predatory behaviour in fossil taxa. *Proceedings of the Royal Society B Biological Sciences*, *272*(1563), 619–625. <https://doi.org/10.1098/rspb.2004.2986>
